# Atomic-Scale Modeling of Water and Ice Behavior on Vibrating Surfaces: Toward the Design of Surface Acoustic Wave Anti-icing and Deicing Systems

**DOI:** 10.1021/acs.langmuir.4c04330

**Published:** 2025-05-01

**Authors:** Tomasz Wejrzanowski, Stefan Jacob, Andreas Winkler, Jaime Delmoral, Ana Borrás, Agustín R. González-Elipe

**Affiliations:** † Faculty of Materials Science and Engineering, Warsaw University of Technology, Woloska 141, 02 507 Warsaw, Poland; ‡ Technology Partners Foundation, Pawinskiego 5A, 02-106 Warsaw, Poland; § Physikalische-Technische Bundesanstalt (PTB), Bundesallee 100, 38116 Braunschweig, Germany; ∥ Leibniz IFW Dresden, SAW Laboratory Saxony, Helmholtz str. 20, 01069 Dresden, Germany; ⊥ Nanotechnology on Surfaces and Plasma Laboratory, Materials Science Institute of Seville (CSIC-US), Américo Vespucio 49, 41092 Seville, Spain

## Abstract

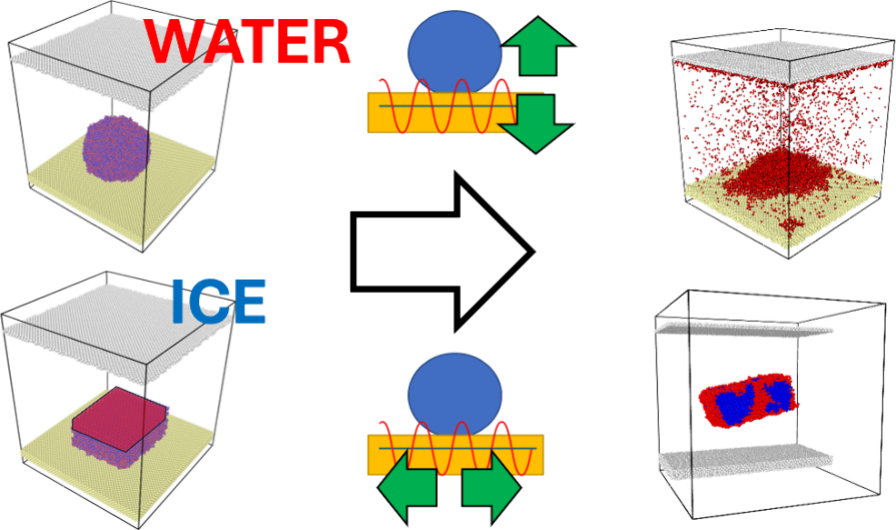

Within these studies, atomic-scale molecular dynamics simulations have been performed to analyze the behavior of water droplets and ice clusters on hydrophilic and hydrophobic surfaces subjected to high-frequency vibrations. The methodology applied herewith aimed at understanding the phenomena governing the anti-icing and deicing process enabled by surface acoustic waves (SAWs). The complex wave propagation was simplified by in-plane and out-of-plane substrate vibrations, which are relevant to the individual longitudinal and transverse components of SAWs. Since the efficiency of such an active system depends on the energy transfer from the vibrating substrate to water or ice, the agents influencing such transfer as well as the accompanying phenomena were studied in detail. Apart from the polarization of the substrate vibrations (in-plane/out-of-plane), the amplitude and frequency of these vibrations were analyzed through atomic-scale modeling. Further, the surface wettability effect was introduced as a critical factor within the simulation of water or ice sitting on the vibrating substrate. The results of these studies allow identification of the different phenomena responsible for water and ice removal from vibrating surfaces depending on the wave amplitude and frequency. The importance of substrate wetting for anti-icing and deicing has also been analyzed and discussed concerning the future design and optimization of SAW-based systems.

## Introduction

Water and/or ice interaction with materials surfaces is of key importance both for life sciences^1,2^ and materials and engineering.^3,4^ The hydrophilicity of most natural material surfaces enables water to be transported over large distances via capillary effects.^5,6^ Similarly, some plants and insects use a particular design of their outer surface to promote water condensation.^7^ Inspired by these observations from nature, specifically designed engineered materials have been proposed to achieve efficient control of wettability. In general, this engineering involves surface treatments aiming at changing both the chemistry and topography of surfaces. Control of surface nanostructure and chemistry is particularly critical to obtaining superhydrophobic surfaces (i.e., water contact angle >150°),^8−11^ as illustrated in the literature by the application of coatings and surface treatments endowing almost full water repellency.^4,12,13^ Experimental studies and numerical simulations have been profusely applied to understand the wetting behavior of surfaces (for example, in terms of the Wenzel and Cassie–Baxter states) as a function of either intrinsic material properties such as surface topography and chemistry or external conditions (temperature and pressure).^14−22^

The icing of materials is a critical issue for many processes and engineering structures, for example, aircraft,^23−25^ wind turbines,^26,27^ and marine structures.^28,29^ In trying to circumvent the problems associated with the icing of surfaces, both passive (i.e., preventing the formation of ice) and active (i.e., provoking the removal of ice) solutions have been proposed. The passive or anti-icing solutions have been widely studied during the last few years, having found a certain but not complete correlation between superhydrophobicity and highly efficient anti-icing response, with the latter in terms of prevention of ice formation and accretion.^30,31^ In fact, to obtain high anti-icing performance, the material surfaces should also hold other important features such as a high capability for delaying freezing or low ice adhesion.^32^ Regarding deicing, a general operational condition is the availability to continuously deliver energy or chemical flow to the surfaces to promote the removal of ice. For example, commercial systems for aircraft are based on thermal heating,^33,34^ pneumatic deice boots,^35,36^ or the application of deicing lubricants.^37^ Drawbacks of most common deicing systems are high energy consumption, low flexibility with regard to materials and environmental compatibility, and limited duration of their effects.

Recently, the use of ultrasound^38,39^ and, more specifically, short-range wave acoustics and surface acoustic waves (SAWs)^40,41^ have been proposed as efficient alternative solutions for deicing or preventing icing (inducing an active anti-icing effect) at relatively low energy costs and high operational flexibility.^42,43^ The interaction of a SAW propagating along a material with water on its surface is a quite mature subject of the investigation with even practical engineering solutions in the market for quite a wide range of applications including microfluidics^44,45^ and lab-on-a-chip systems.^46,47^ However, the study of the effects of the SAW with accreted ice is still in its infancy and lacks fundamental knowledge about the interaction of ice with the atomic surface oscillations typical of these waves, which might provide critical information about the most efficient solutions out of several possibilities for operational surface and bulk acoustic waves. This and similar processes such as fluid nebulization, surface wetting, and ice melting are fast processes that are initiated locally and as such are difficult to characterize with sufficient precision. In this context, atomic-scale simulation becomes a useful tool to simulate water and ice behavior on the surface of materials. It has been used to understand the chemical and physical bonding of water molecules to various surfaces.^48−53^ Also, the freezing delay has been recently analyzed for nanoscale systems,^54^ aiming at designing superhydrophobic and icephobic coatings and surfaces. Recently, a few theoretical works have also addressed the water behavior on vibrating substrates activated by SAWs.^55,56^ However, to the best of our knowledge, no atomic simulation works about the interaction of SAW with ice exist so far. Previous reports about the interaction of SAWs with water droplets suggested that the interaction is based on the transfer of the kinetic energy associated with the vibrating atoms of the substrate to the water molecules in contact with it. The energy-transfer interaction will be modeled in this work, assuming the effect of a vibrating rigid substrate of ordered atoms at the interface with either water or ice.

In a standard SAW device, the electromechanical excitation is typically induced by a transducer formed by a comb-shaped interdigitated electrode (interdigital transducer, IDT) deposited on a piezoelectric substrate or a piezoelectric thin film. The wave generated in such a way propagates along the surface of the substrate and efficiently transfers the energy to water (or ice) on the surface.^57^ Rayleigh waves are the most common SAW mode for actuation and energy transfer into a half space atop. Rayleigh waves present a characteristic frequency, wavelength, and direction that depend on IDT architecture and orientation as well as piezoelectric material properties and are characterized by a sagittal polarization, i.e., with in-plane (longitudinal) and out-of-plane (surface-normal) components.

This work aims to gain knowledge about the fundamental mechanisms involved in the transfer of energy between a vibrating surface and an ice aggregate placed on its surface. For comparison, a similar approach is applied to a water droplet. The objective is to unravel some of the basic mechanisms governing water and ice removal and the effect of such processes on the wetting state of the material surfaces (i.e., their hydrophobic or hydrophilic character). An additional goal is to determine basic SAW characteristics responsible for water or ice activation, mainly the effect of the in-plane and out-of-plane components of the wave on the effectiveness of the deicing and water removing processes. The results show that the effectiveness of water removal and ice melting varies differently with the amplitude, frequency, and horizontal/vertical components of the atomic surface vibrations and, in general, is favored by the hydrophobic character of the surface state.

## Materials and Methods

Herein we propose to employ a rigid substrate vibrating either horizontally or vertically as a model system to simulate by molecular dynamics (MD) the effect of atomic surface vibrations typical of SAWs on the excitation of a water droplet or an ice cluster deposited on their surface. We assume that these substrate vibrations and the mechanical interaction occurring with the droplet/ice cluster placed on their surfaces reproduce the mechanical energy transfer taking place in real systems between the SAW-activated substrates and the droplets/ice cluster on their surfaces. Concretely and as pointed out in the Introduction, SAW has in-plane and out-of-plane components that are simulated in this paper by the vertical and horizontal vibrational modes of the substrate, providing a way to estimate the most efficient wave mode for energy transfer to either water or ice.

Atomic-scale simulations of the behavior of a water droplet and ice cluster on a vibrating substrate were performed using classical molecular dynamics (MD) calculations implemented in the LAMMPS software. The core code for water dynamics in LAMMPS was adopted from ref (55) and later advanced to account for ice cluster modeling. Parallel computations were carried out using a high-performance in-house computer cluster under the MPI protocol.

Simple Newton’s equations of motion are solved within the MD simulations
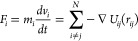
1where *F*_*i*_, *m*_*i*_, and *v*_*i*_ are the force, mass, and velocity of each particular atom (molecule) under consideration. *N* indicates the number of atoms, and *t* is time. *U* is defined as the interatomic potential between two atoms *i* and *j* separated by *r*_*ij*_. In these simulations, the Leonard-Jones and Coulomb potentials have been used
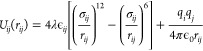
2where σ is the distance at which the interatomic potential is zero, ϵ is the depth of the potential well, *q* is the charge of the atomic site, and *ϵ*_0_ is the vacuum permittivity. A λ parameter allows us to tune the substrate–liquid interaction strength and hence to define the static contact angle, θ. It has been adjusted from superhydrophobic (λ = 0.15, θ ≈ 160°) to hydrophilic (λ = 1, θ < 20°).

The first term on the right side of eq 2 represents the intermolecular forces, and the second term represents the electrostatic contributions. Polar water molecules are modeled using the rigid four-site TIP4P/2005 model;^58^ this consists of one oxygen (O) site, two charged hydrogen (H) sites, and one massless charged (M) site located along the bisector of the hydrogen atoms, at a distance of 0.1546 Å from the oxygen atom. The internal geometry of the water molecule is constrained by specifying a fixed O–H bond distance (0.9572 Å) and H–O–H angle (104.52°); this structure is maintained using the SHAKE algorithm.^59^ The droplet-supporting substrate consists of 6 layers of Pt metal atoms in an FCC lattice, with a lattice constant of 3.92 Å. All of the interatomic potential parameters are listed in Table 1.

**Table 1:** Interatomic Potential Parameters and Atomic Masses (*m*_a_) for Sites in TIP4P/2005 Water Atom/Molecules (H, O, and M) and Substrate Platinum (Pt) Atoms

Site	ϵ (kJ/mol)	σ (Å)	*q* (e)	*m* (u)
H	0	0	0.5242	1.008
O	0.774	3.1589	0	15.9994
M	0	0	–1.0484	0
Pt	4.18	2.471	0	195.084

**Figure 1:**
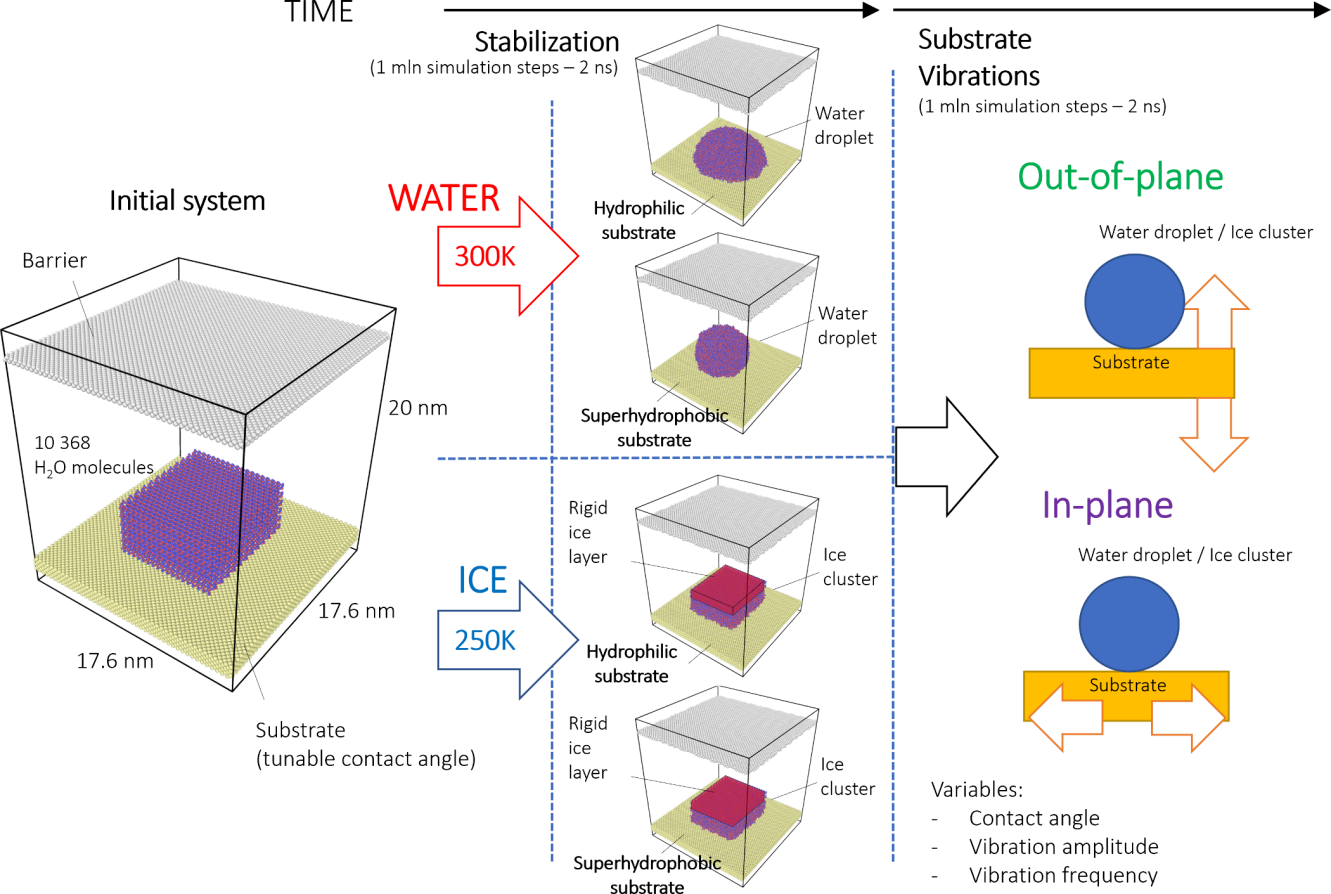
Schematic illustration of the molecular dynamics simulations from the initial system (left) to the stabilization at two different temperatures (middle) including hydrophilic (WCA ≈ 20°) and superhydrophobic (WCA ≈ 160°) surfaces and variables for out-of-plane and in-plane vibrations (right).

As shown in Figure 1, the initial layout used for calculations consists of a cluster of ordered water molecules located on the atomically flat substrate. The domain boundaries are set to be periodic in every direction, and a superhydrophilic barrier is positioned far above the substrate to collect atomized molecules and prevent them from leaving the domain via the top boundary and reentering through the bottom boundary. The values of σ, ϵ, and atomic mass *m* for the substrate/barrier atoms are derived from those of platinum, which is selected as the substrate with no water–substrate chemical bonding.

The MD simulation system in Figure 1 comprises a cluster of 10368 water molecules (hexagonal crystal). It is equilibrated for 2 ns at 300 and 250 K for the stabilization of a water droplet and an ice cluster, respectively. It is reported that for the infinite ice crystal, the melting simulated with the use of the TIP4P/2005 model starts at 250 K rather than at 273.1 K.^60^ Also, for the spherical water cluster with 7931 molecules the melting point drops down to 237.5 K.^61^ In order to keep the ice crytal structure during equilibration, the outermost top 4 layers were grouped into a rigid body, thus avoiding surface melting effects described above for water and also typical for other nanoobjects.^62,63^

Following equilibration, the simulation is run for a period of 2 ns (with the time step of 2 fs), during which the substrate is oscillated either surface-normal (out-of-plane) or horizontally (in-plane) at frequencies of between 50 and 200 GHz and for a range of amplitudes between 0.1 and 1 nm. Substrate wettability values of 20° (simulating hydrophilicity) and 160° (simulating superhydrophobicity) have been considered for the calculations. Temperature control is applied only to the water/ice molecules during stabilization (NVT ensemble) and later released when the vibrations start. The substrate and barrier molecules are coupled to a Berendsen thermostat to maintain the surface at a constant temperature (300 K for water and 250 K for ice cases, respectively).

OVITO software was used to visualize the system evolution. To differentiate between ice and water molecules, the oxygen atoms were identified as belonging or not to the hexagonal-type structure characteristic of the initial arrangement of water molecules. This classification tool has been implemented in the OVITO program.^64^

The OVITO software was also used for the identification of the atomization/boiling/lift-off process. Usually, if lift-off occurred, it was observed at a relatively early stage of simulation and the process was rapid. To distinguish the atomization and boiling, a cluster size analysis tool available in AVIZO software was used. This algorithm decomposes a particle system into disconnected sets of particles (clusters) based on a local neighboring criterion. The neighboring criterion is based on the distance between the particles. The particle belongs to the cluster if its distance to the nearest neighbor is lower than the cutoff distance. In our calculations, we took into consideration only the oxygen atom distance, and the cutoff distance was set to 3.2 Å. This value was based on the radial distribution function (RDF) for oxygen atoms in water. We found that the oxygen atoms distance in the liquid water droplet does not change significantly with the temperature and varies at around 2.7–2.9 Å (ref (65)).

**Figure 2:**
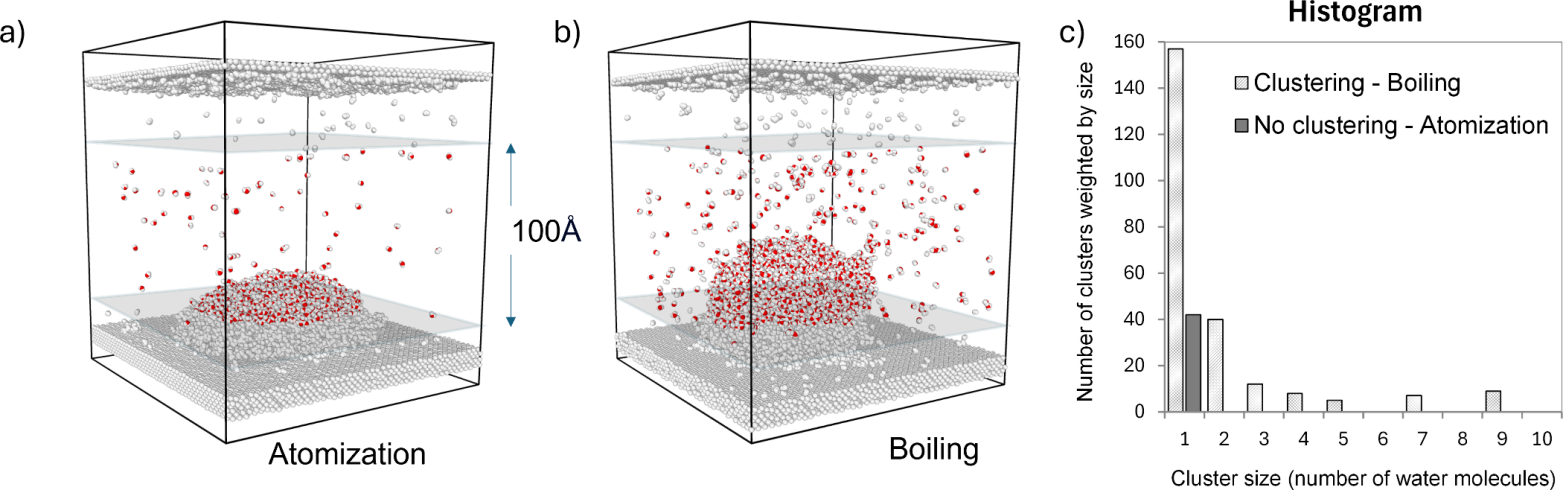
Schematic illustration of the identification of the atomization/boiling process. Example a) atomization and b) boiling snapshots. c) Histogram of cluster size for atomization/boiling.

For the cluster identification, the area 100 Å high over the surface (10 Å over the surface) was selected. The process is boiling if clusters with a size larger than 2 water molecules appear in the histogram. This has been presented schematically in Figure 2.

## Results and Discussion

### Vibrational Excitation of the Water Droplet

**Figure 3:**
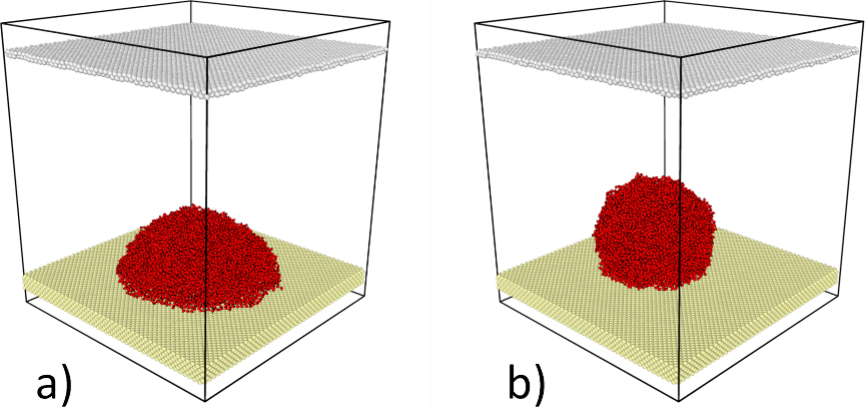
Atomic structure after 2 ns stabilization for a) hydrophilic and b) hydrophobic surface states.

The initial ice cluster shown in Figure 1 was molten at 300 K, and the water droplet was stabilized at this temperature for 2 ns. Two different substrates were used in the calculations to simulate the effect of surface wettability. Atomic structures after 2 ns stabilization at 300 K are shown in Figure 3.

After stabilization of the water droplet on the surface, the substrate was subjected to two types of vibrations: horizontal (in-plane) and vertical (out-of-plane). The water droplet behavior was observed for 2 ns of simulation time for various values of amplitude and frequency of the substrate vibrations. Specific values of these parameters were varied during the calculations to identify the transition limits between various physical phenomena. Three types of processes were distinguished, namely, atomization, boiling, and lift-off. Those processes have been also identified by other authors.^55,56^ Atomization is found as a single or few water molecules departing from the water droplet. Boiling, which requires a higher energy, has been identified when a collective group of molecules depart from the water droplet. The lift-off process characterizes the entire detachment of the water droplet from the surface.

Specific simulations are signaled and selected for the relevant representations in Figure 5.

**Figure 4:**
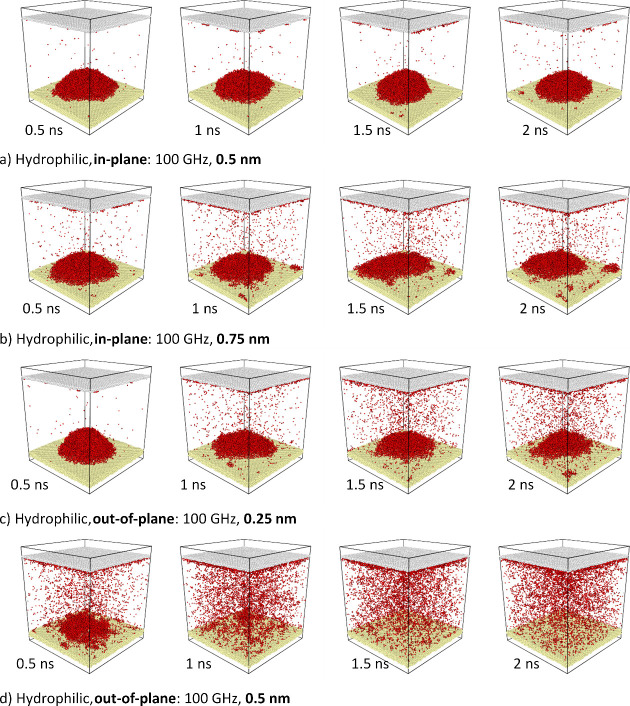
Series of snapshots showing the dynamics of a water droplet on a hydrophilic substrate subjected to a,b) in-plane and c,d) out-of-plane vibrations at 100 GHz frequency and various amplitudes: a) 0.5, b) 0.75, c) 0.25, and d) 0.5 nm.

The initial stage of water droplet dynamics indicates that the wetting angle progressively decreases with time (see, in particular, Figure 4b,c). This phenomenon has been found experimentally for water droplets activated with SAWs, and it is called the “acoustowetting effect”.^66−68^

For a better comparison of the process efficiencies, Figure 4 shows a series of snapshots of the droplet time evolution for a hydrophilic surface. These snapshots support the fact that for hydrophilic surfaces the atomization/boiling process is much faster for out-of-plane vibrations (compare the evolution of snapshots in Figure 4a,d).

**Figure 5:**
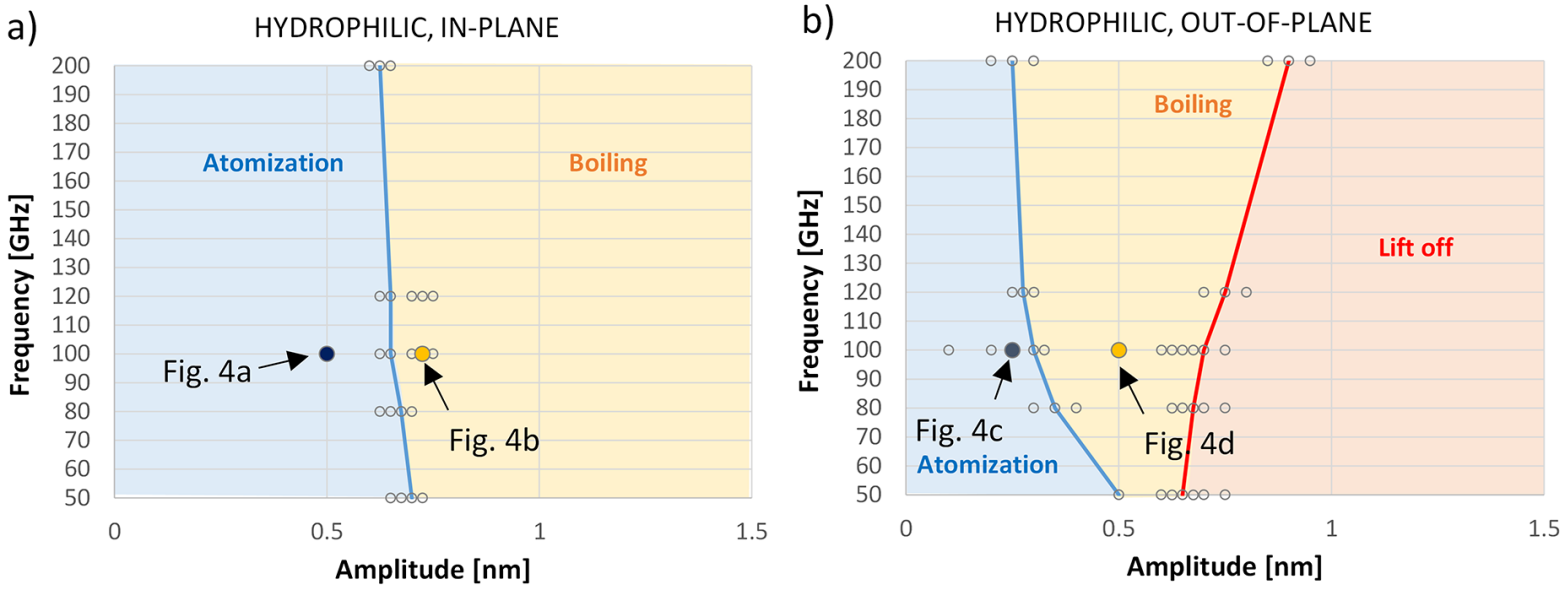
Maps of excitation phenomena for a water droplet sitting on a hydrophilic substrate subjected to a) in-plane and b) out-of-plane vibrations as a function of specific amplitude and frequency values.

The map of the processes taking place in a hydrophilic system is presented in Figure 5. In the plots, the small circles represent calculations for specific couples of amplitude and frequency values, while the lines and background colors identify the zones where the different phenomena (atomization, boiling, and lift-off) take place.

These plots show that the amplitude of the substrate vibrations and not the frequency is the critical variable to activate the water droplet. It is also apparent that for the same amplitude and frequency, out-of-plane vibrations are more effective than in-plane vibrations to activate atomization and boiling phenomena. For large amplitude values, droplet lift-off is observed only for out-of-plane vibrations and not for in-plane ones.

The analysis of the temporal evolution of water droplet behavior, along with further subsequent quantitative calculations, indicates that energy transfer on hydrophilic substrates is more efficient when vibrations are applied in the out-of-plane direction rather than in-plane. This may explain the stronger frequency dependence observed for the transition from atomization to boiling, as shown in Figure 5a compared to Figure 5b.

**Figure 6:**
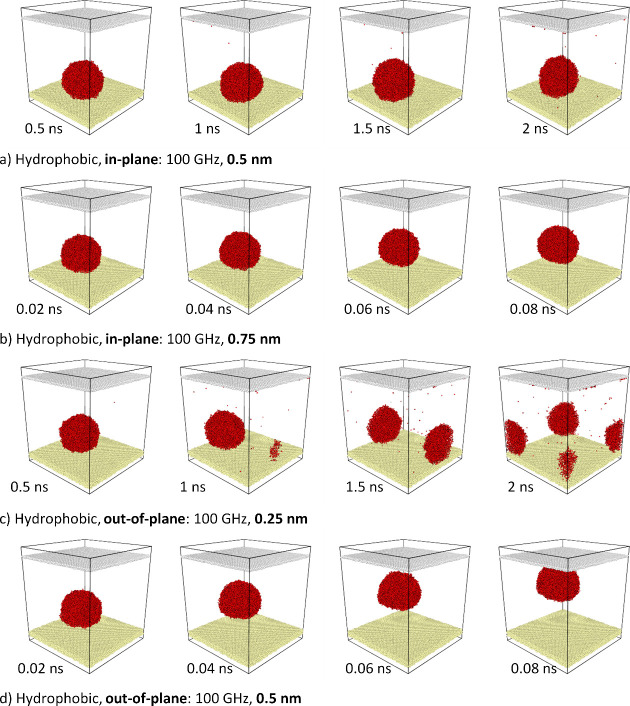
Series of snapshots showing the dynamics of a water droplet on a superhydrophobic substrate subjected to a,b) in-plane and c,d) out-of-plane vibrations at 100 GHz frequency and various amplitudes: a) 0.5, b) 0.75, c) 0.25, and d) 0.5 nm.

Significantly, different behavior was observed for hydrophobic surfaces. As observed in Figure 6, the atomization process is in this case less pronounced and the dominant energy-transfer mechanism is through droplet lift-off, even for relatively low amplitude values.

This is more clearly seen in the series of snapshots for hydrophobic surfaces presented in Figure 6. They show that low-energy vibrations (low amplitude and low frequency) give rise to a very slow and limited atomization process (Figure 6a). Meanwhile, for higher vibrational amplitudes, the water droplet starts to move over the surface (Figure 6b). The application of larger vibrations results in the entire droplet detaching from the surface (lift-off process) (Figure 6b,d).

**Figure 7:**
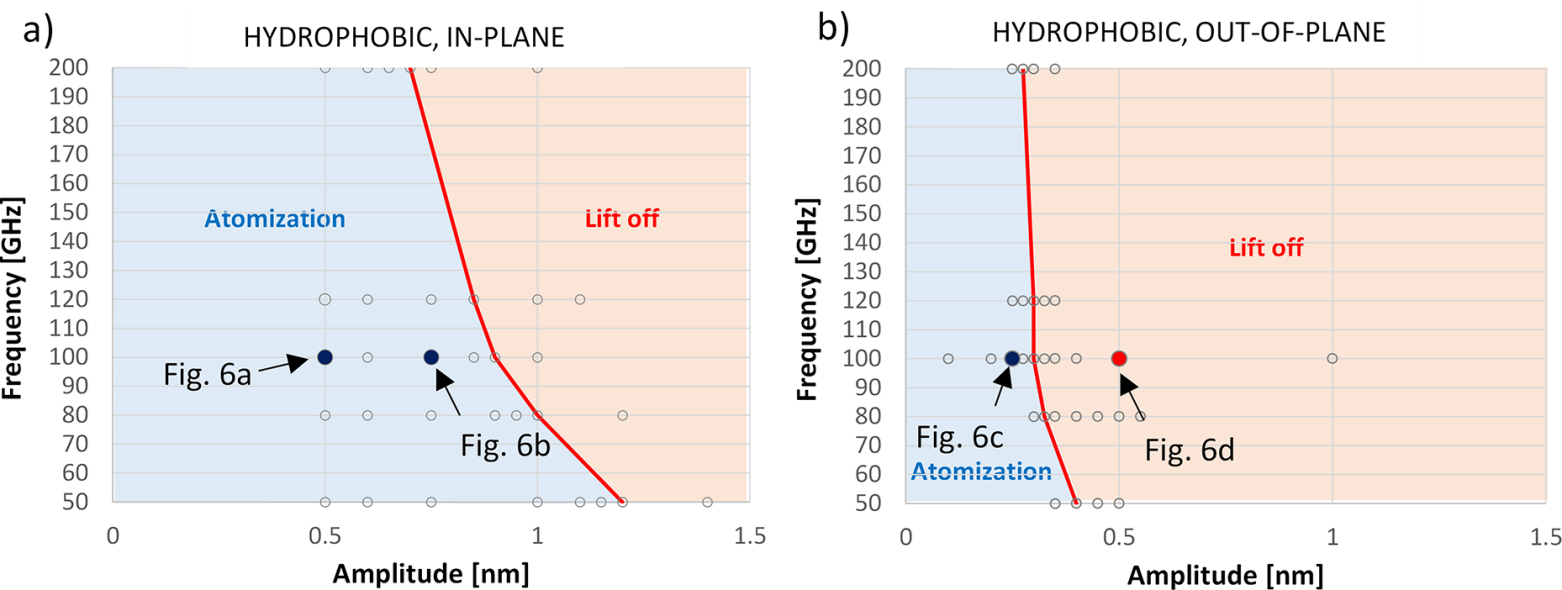
Maps of excitation phenomena for a water droplet sitting on a superhydrophobic substrate subjected to a) in-plane and b) out-of-plane vibrations as a function of specific amplitude and frequency values.

**Figure 8:**
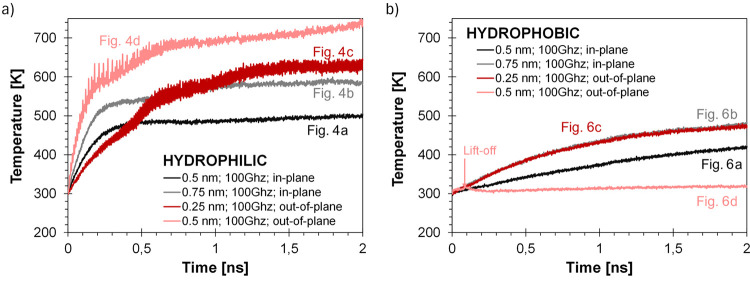
Water droplet temperature changes over time for a) hydrophilic and b) hydrophobic substrates.

In contrast, the behavior on hydrophobic substrates differs significantly from that on hydrophilic. Due to the limited contact area between the droplet and the surface, restricted primarily to the time the droplet remains in contact, the energy-transfer dynamics change. Our temporal analysis and calculations reveal that out-of-plane vibrations facilitate droplet lift-off more readily. Conversely, during in-plane vibrations, the droplet remains on the surface for a longer duration, allowing for more sustained energy transfer. This suggests that the influence of frequency on in-plane vibrations is more pronounced for hydrophobic surfaces compared to the out-of-plane mode, as shown in Figure 7a compared to Figure 7b. In Figure 8, the temperature change over time for the water droplet under the conditions representing various mechanisms of the energy transfer through atomization/boiling/lift-off is presented. Each particular temperature profile corresponds to the 3D atomic system state snapshots (Figures 4 and 6). The temperature profiles give deeper insights into the mechanism of energy transfer from the vibrating substrate to the water droplet.

In order to better understand the temperature (kinetic energy) distribution within water/ice molecules, we utilized OVITO software. Using the atomic mass, *m*_a_, and velocity, *v*_a_, we calculated the kinetic energy of each atom, *E*_ka_. From this, we determined the corresponding “temperature” of individual atoms, *T*_a_. While temperature is fundamentally a statistical measure that is meaningful for large ensembles of atoms, we believe that presenting the “temperature of individual atoms” provides a clearer and more consistent comparison with the temperature profiles used elsewhere in the article. Therefore, we presented this quantity rather than reported the individual kinetic energies. We employed the following equations for our calculations
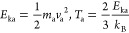
3where *k*_B_ is the Boltzmann constant.

If the energy is calculated in eV, then *T*_a_ ≈ 11604*E*_ka_

**Figure 9:**
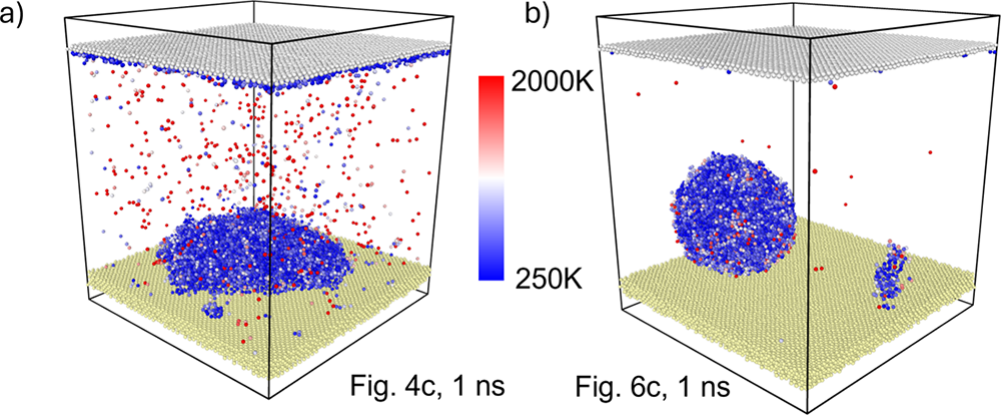
Temperature distribution within the water region for selected representative cases: a) hydrophilic, out of plane, 100 Ghz, 0.25 nm, 1 ns, see also Figure 4c; b) hydrophobic, out of plane, 100 Ghz, 0.25 nm, 1 ns, see also Figure 6c.

These calculations are particularly valuable, as they reveal that the kinetic energy (or “temperature”) is not uniformly distributed among the water molecules. As illustrated in Figure 9, the initial water droplet exhibits relatively low temperature while the atoms departing from the droplet possess significantly higher kinetic energy.

The temperature profiles in Figure 8 represent the temperature of the entire water region, calculated based on the total kinetic energy of all atoms, including both those within the initial droplet and the molecules that have departed from it. This explains the relatively high temperature in some cases. Notably, similar observations were reported by Pillai et al.^55^

The maximum energy, *E*_max_, generated from the vibrating substrate can be defined irrespective of the type of vibration (in-plane or out-of-plane) as
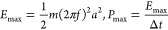
4where *m* is the mass of the substrate, *f* is the frequency, and *a* is the amplitude of vibrations. *P*_max_ indicates the maximum power induced by the vibrating substrate for the duration of time Δ*t*.

However, only a part of this energy is transferred to the water droplet mostly due to the interaction between the vibrating substrate and the water droplet, which depends on factors like the contact area, adhesion energy end dissipation of energy in the form of heat, and/or droplet deformation and motion. Furthermore, the nature of the vibration, such as the mode (in-plane or out-of-plane) and its resonance with the droplet, also significantly impacts the energy-transfer efficiency. The droplet’s dynamics, including deformation, oscillation, and eventual detachment or movement, are governed by these factors, leading to a nonideal energy transfer from the substrate to the droplet.

In the in-plane mode, the substrate vibrates within its surface plane (horizontal), causing shear forces at the interface between the substrate and the water droplet. These forces lead to localized heating in the droplet as the energy is transferred from the vibrating substrate to the droplet through the contact area. The heating can be intense enough to cause a rise in the temperature of the droplet, potentially leading to atomization or boiling.

The adhesion energy and contact area between the droplet and the substrate also play critical roles. If the substrate/water has a low adhesion force (hydrophobic surface), then the shear forces from the in-plane vibrations may be more likely to overcome the adhesion and cause lift-off.

In the out-of-plane mode, the substrate vibrates perpendicular to its surface (vertical), causing compression-tensile water droplet deformation. Compression and expansion (as seen in out-of-plane vibrations) affect the entire droplet and can lead to larger energy dissipation compared with shear forces, which act more locally and may not heat the entire droplet effectively. If the vibration power becomes sufficiently large (i.e., the amplitude and frequency of vibration are high enough), then the forces acting on the droplet may overcome the adhesive forces holding it to the substrate. This can lead to the transfer of the substrate energy to the kinetic motion of the droplet, leading to its lifting off from the surface.

Energy transfer from the vibrating substrate to the droplet can be the sum of the energy of droplet heating and the kinetic energy of the droplet motion. The energy consumed for heating, *E*_h_, can be derived from the following equation
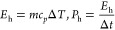
5where *m* is the mass of the water droplet, *c*_*p*_ is the specific heat, and Δ*T* is the temperature change induced by the energy transferred. *P*_h_ indicates the power transferred for the duration of time Δ*t*.

The kinetic energy, *E*_k_, of the droplet motion induced by the energy transfer from the vibrating surface can be estimated by the classical formula
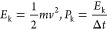
6where again *m* is the mass of the water droplet and *v* is the droplet velocity. *P*_k_ indicates the power transferred for the duration of time Δ*t*.

For the hydrophilic case (Figure 8a) where the adhesion forces are strong and the contact area is large, we may observe that the substrate vibrations, both in-plane and out-of-plane, result in water droplet heating only. For the in-plane vibrations, the droplet temperature increases to some certain value and then stabilizes. In the out-of-plane mode, the transfer of energy to the water droplet is much more efficient, resulting in a faster droplet temperature increase and more rapid evaporation phenomena consequently.

The hydrophobic case (Figure 8b) is significantly different. It can be observed that for the same substrate vibration conditions the droplet heats to a lower temperature than in the hydrophilic case. The atomization process is consequently low pronounced. For larger vibration amplitudes, the droplet starts to move over the surface (Figure 6b,c). If the vibrations are very high (and preferably out-of-plane), then the droplet overcomes the adhesion forces and detachs from the surface. In this case, the temperature increase of the droplet is low and later decreases due to the fact that it touches the top barrier which is stabilized at 300 K.

Based on eqs 4–6, we have quantitatively compared the energy transfer in the hydrophobic and hydrophilic water systems subjected to both in-plane and out-of-plane vibrations at the same frequency and amplitude (100 GHz and 0.5 nm). The hydrophilic in-plane system is shown in Figure 4a, and the hydrophilic out-of-plane system is shown in Figure 4d. The hydrophobic in-plane and out-of-plane systems are presented in Figure 6a,d, respectively.

For the hydrophilic systems (Figure 4a,d), we assumed that energy transfer occurs solely via heating of the water droplet. In contrast, for the hydrophobic systems, particularly that shown in Figure 6a, a mixed mechanism is considered. For the system illustrated in Figure 6d, energy transfer from the vibrating substrate to the droplet is calculated based on the kinetic energy associated with droplet lift-off, using eq 6. The average velocity of the droplet was obtained using OVITO, calculated as the total distance traveled over time. For lifting cases, this velocity was determined up to the point at which the droplet made contact with the upper boundary.

To better compare the systems at 1 ns, we used the power transfer (energy per unit time) as the primary metric. For the case where the droplet lifts off the substrate (Figure 6d), the time interval for power calculation was limited to the duration of droplet–substrate contact (0.01 ns).

According to eq 4, the maximum power supplied by the vibrating substrate at 100 GHz with 0.5 nm amplitude over 1 ns is 780 nW. Using eq 5, the power transferred to the water droplet on the hydrophilic substrate with in-plane vibration (Figure 4a) is 240 nW, while for the out-of-plane case (Figure 4d), it is 510 nW. These correspond to temperature increases of 187 and 395 K, respectively (Figure 8a). This indicates quantitatively that out-of-plane vibrations are more effective than in-plane vibrations in terms of energy transfer.

For hydrophobic systems, the power transferred to the droplet in the in-plane mode (Figure 6a) is 109 nW (95 nW from heating and 14 nW from lateral motion, based on eqs 5 and 6). In the out-of-plane mode (Figure 6d), the power is 155 nW, based on a traveled distance of 10 nm in 0.1 ns with droplet–substrate contact lasting 0.01 ns. The temperature increase is negligible in this case, so only eq 6 applies (Figure 8b). These results indicate quantitatively that energy transfer from the vibrating substrate to the water droplet is generally weaker for hydrophobic systems compared to hydrophilic ones. However, significantly less power is required to induce droplet motion or lift-off than to achieve full evaporation. It is worth noting that for larger droplets, gravity (neglected in our simulations) may play a more significant role and would likely increase the power required for lift-off.

### Vibrational Excitation of the Ice Cluster

Unlike the activation of water droplets with SAWs, where ample literature accounts well for the activation mechanism, only a couple of recent papers^40,41,69^ have addressed the analysis of the mechanism of ice activation and its eventual melting. The current atomic-scale analysis pretends to determine the parameters and conditions as being more favorable for the energy transfer from a vibrating substrate to ice. In particular, information will be retrieved on whether excitation via in-plane or out-of-plane vibrations (simulating the effect of normal and shear components of SAWs) is more efficient for deicing substrates with specific wetting behavior (hydrophobic and hydrophilic). In the case of ice clusters, a significantly different response is expected when compared with the behavior of the water droplets. Ice contrary to water is difficult to deform plastically, and the strain induced by moving the substrate causes relatively large stresses. Those stresses might be slightly reduced when a thin water layer between ice and the substrate is present.

**Figure 10:**
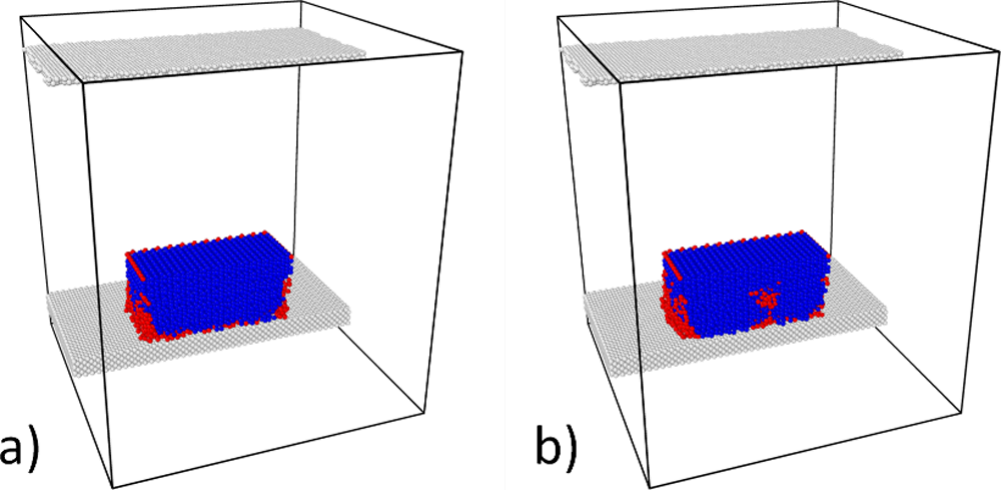
Cross section through the simulated atomic system representing an ice cluster after stabilization for 2 ns at 250 K on a) hydrophilic and b) hydrophobic surfaces. Red – water; blue – ice.

Before the analysis of the activation of ice, it is interesting to visualize the initial state of the ice cluster. Figure 10 presents the ice cluster utilized for the simulation after its stabilization at 250 K for 2 ns. This stabilization was done on the hydrophilic and superhydrophobic surfaces. On the hydrophilic substrate, the reported snapshot reveals the formation of a continuous thin atomic water layer between ice and the substrate. On the hydrophobic substrate, some water molecules also form liquid nuclei inside the ice cluster. This has also been observed by other authors.^43,70^

Such an effect is congruent with the fact that a thin water layer appears at the ice/substrate interface. The shear forces induced by in-plane vibrations may easily deform the water layer without transferring the deformation to ice. However, the out-of-plane vibrations cannot be damped by the water atomic layer at the interface, and especially for larger amplitudes, kinetic energy can be effectively transferred to ice.

**Figure 11:**
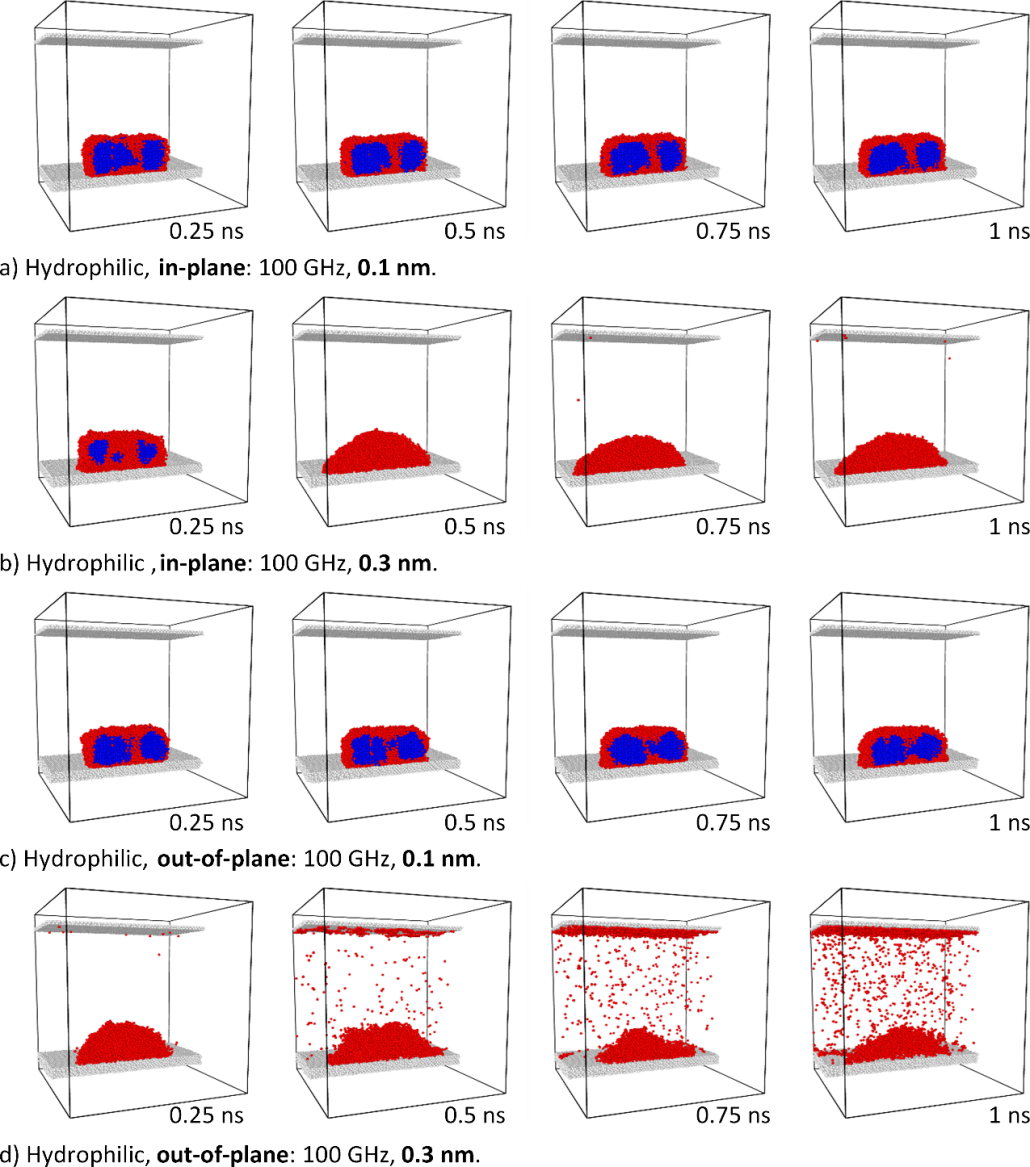
Dynamics of the ice cluster on the hydrophilic substrate subjected to a,b) in-plane and c,d) out-of-plane vibrations of 100 GHz frequency and amplitudes of a,c) 0.1 nm and b,d) 0.3 nm.

**Figure 12:**
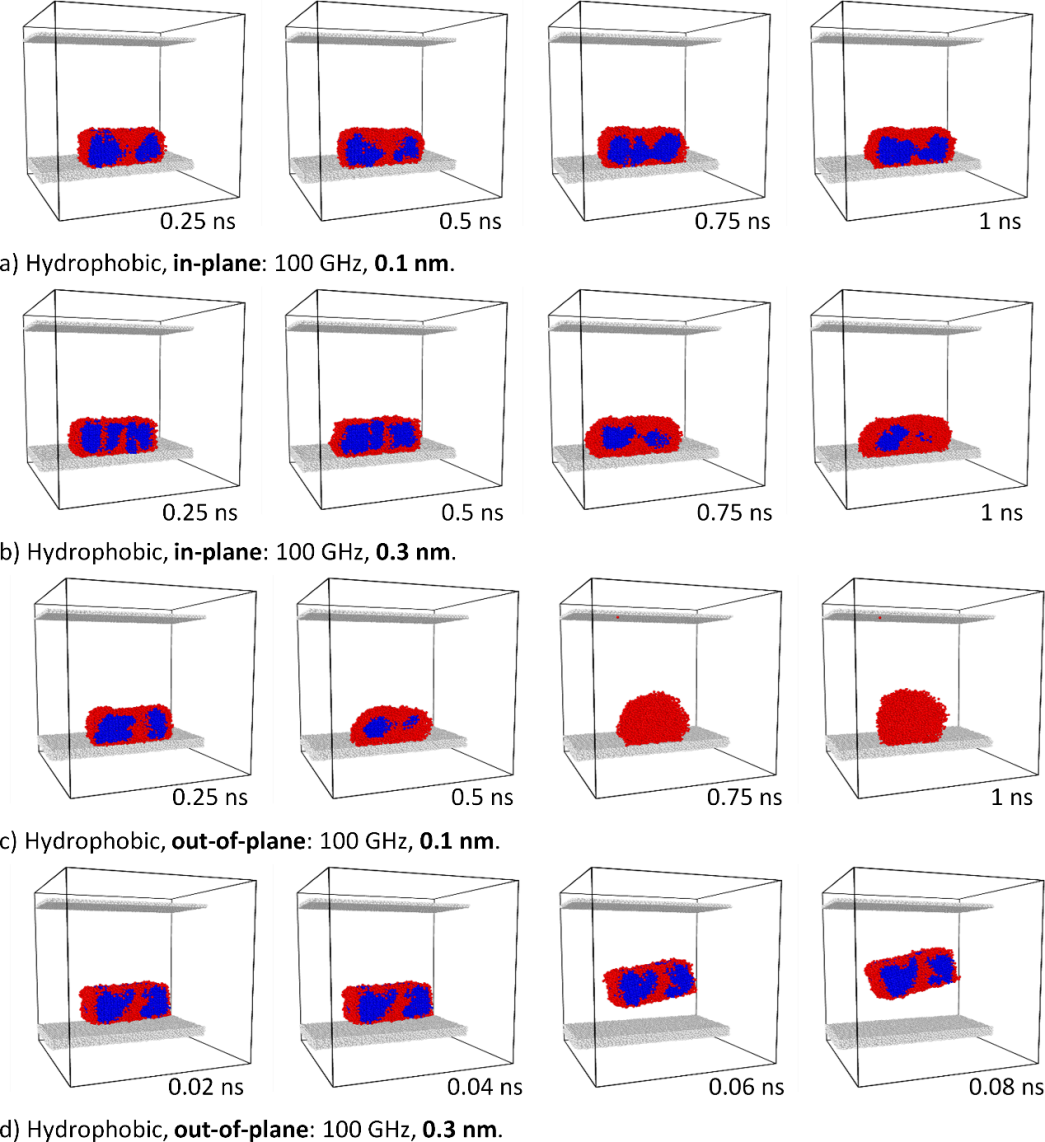
Dynamics of the ice cluster on the hydrophobic substrate subjected to a,b) in-plane and c,d) out-of-plane vibrations of 100 GHz frequency and amplitudes of a,c) 0.1 nm and b,d) 0.3 nm.

Starting from these two initial stabilized states at 250 K, the simulation of ice cluster behavior upon activation by the vibrating substrate was carried out using conditions similar to those applied for water droplet activation. Thus, in-plane and out-of-plane vibrations were applied to the ice clusters for various frequencies and amplitudes. The obtained molecular dynamics simulation results have shown that the ice cluster may start to melt, evaporate, or become detached from the surface depending on the energy (i.e., the amplitude of vibration) provided by the vibrating substrate as well as on its wetting angle. A set of significant results have been gathered in Figures 11 and 12.

The first analysis in Figure 11 refers to the hydrophilic substrate. It has been found that for low-amplitude vibrations (0.1 nm, see Figure 11a,c) the ice cluster is relatively stable both for in-plane and out-of-plane substrate motions. However, significant differences have been observed for larger amplitudes (0.3 nm, Figure 11b,d), for which out-of-plane vibrations resulted in much faster ice cluster melting and subsequent intense water evaporation.

An analysis of the ice cluster behavior on a hydrophobic substrate is shown in Figure 12. Unlike the hydrophilic system, what is most remarkable in this case is that larger in-plane vibrations do not result in rapid and complete melting of the ice aggregate (compare Figures 11b and 12b) but in partial melting of the outer zones of the ice aggregate where an inner ice core remains after the excitation. This result is in good agreement with the experimental evidence gathered in refs (40) and (41), the first one dealing with surface acoustic waves and the second one dealing with plate acoustic waves. In both articles, the immediate formation of the liquid water interface between the substrate and the ice has been revealed as part of the deicing mechanism. Results in Figure 12 also indicate that in-plane vibrations are less effective for the melting of ice (i.e., to induce complete deicing) on hydrophobic than on hydrophilic surfaces.

In further simulations, out-of-plane vibrations were applied to the ice cluster sitting on the hydrophobic substrate (Figure 12). In this case, for low-amplitude vibrations, ice melting occurred (Figure 12c), and for larger amplitudes, the ice cluster lifts off from the surface (Figure 12d). Similar behavior has been observed recently during experimental studies, where small ice clusters are directly removed from the SAW excited surface without melting. More extended results are going to be presented as a separate paper.

These simulations of the effect of a vibrating substrate on an ice agglomerate support that to attain effective deicing (either melting of ice or detachment of ice from the substrate) surfaces should be preferably hydrophobic and that induced SAW atomic vibrations of the substrate should be tuned to amplify the normal (out-of-plane) wave component. Interestingly, simulations show that for the hydrophilic surfaces the out-of-plane vibrations are also more effective than the in-plane vibrations, particularly to induce water droplet evaporation (Figure 4), while the melting of ice is less effective with this excitation mode (Figure 11).

**Figure 13:**
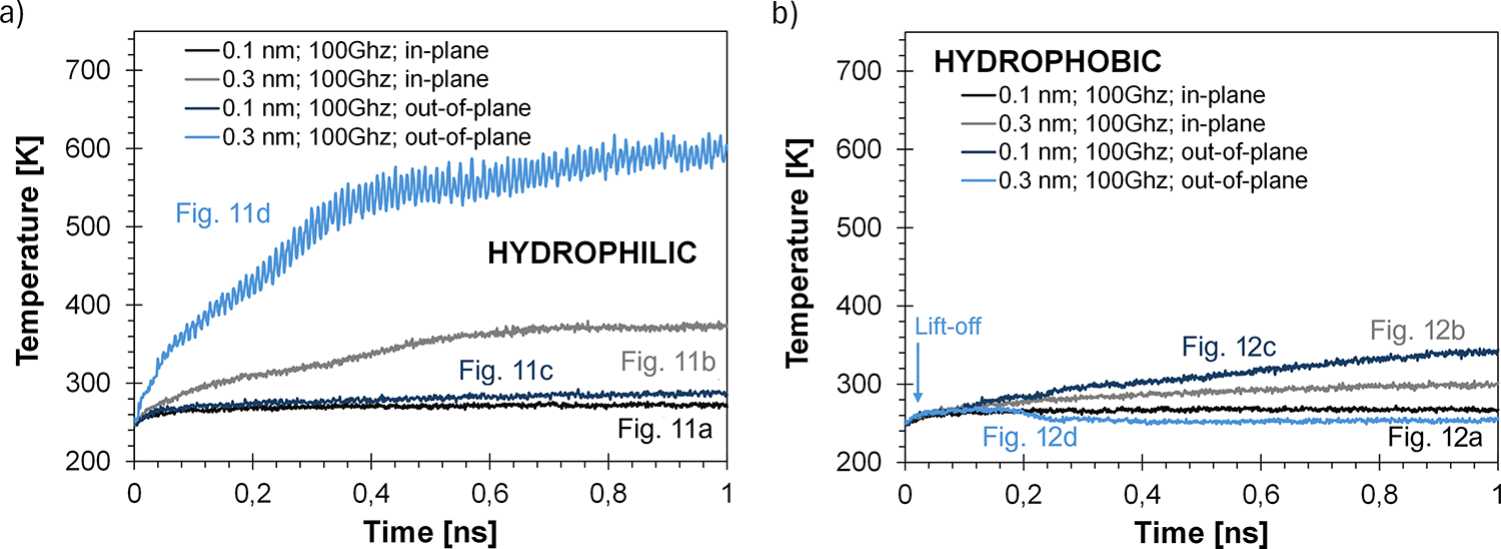
Ice cluster temperature changes over time for a) hydrophilic and b) hydrophobic substrates.

In the case of ice, similar considerations might be applied, as was done for water. The transfer of energy from the vibrating substrate can also be led by ice cluster heating (Figure 13), resulting in melting and then evaporation, or directly by the kinetic energy observed as the cluster motion (lift-off).

**Figure 14:**
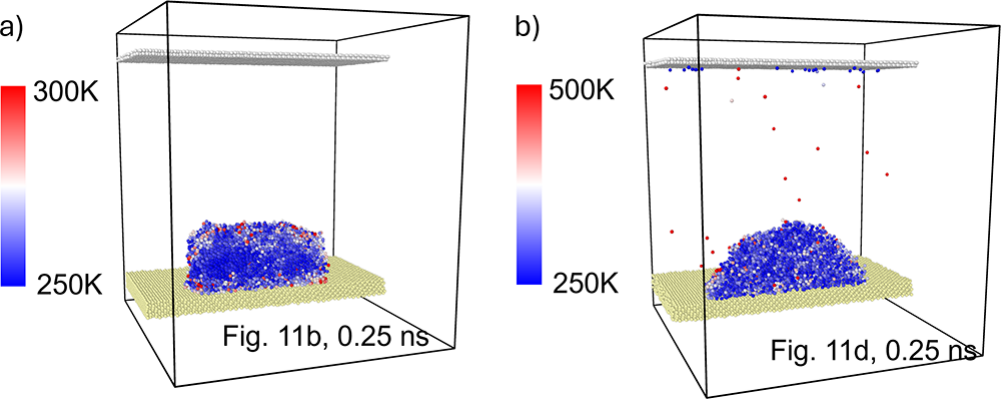
Temperature distribution within the ice/water region for selected representative cases: a) hydrophilic, in-plane, 100 GHz, 0.3 nm, 0.25 ns, see also Figure 11b and b) hydrophilic, out-of-plane, 100 GHz, 0.3 nm, 0.25 ns, see also Figure 11d.

In order to reveal the temperature distribution in the ice cluster system, similar calculations were performed as for the water droplet. The crystalline ice region maintains a relatively low temperature (∼250 K) until the onset of melting (Figure 14). Atoms departing from the droplet exhibit significantly higher kinetic energy (or “temperature”) compared with those remaining within the ice structure.

By an analysis of the behavior of an ice cluster sitting on the hydrophobic surface vibrating with an out-of-plane mode (Figure 12d), one may see that the lift-off of the ice cluster occurs at a lower amplitude than is observed for water droplets (Figure 6d). This can be explained by the fact that the ice is harder to deform than water and that there is no dumping effect which consumes part of the energy.

Analogous energy-transfer calculations were performed for the ice clusters, following the same methodology as that for the water droplet. For this, hydrophilic and hydrophobic systems were evaluated with both in-plane and out-of-plane vibrations at the same frequency and amplitude (100 GHz and 0.3 nm). The hydrophilic in-plane system is shown in Figure 11b, and the hydrophobic out-of-plane system is shown in Figure 12d.

According to eq 4, the maximum power generated by the vibrating substrate under these conditions is 280 nW. Using eq 5, the power transferred to the ice cluster in the hydrophilic in-plane system (Figure 11b) is 156 nW, indicating an energy-transfer efficiency of ∼56%. This is notably higher than the ∼31% efficiency observed for the water droplet under similar conditions (Figure 4a).

For the hydrophobic system with out-of-plane vibrations (Figure 12d), the transferred power is 79 nW, based on a displacement of 10 nm in 0.14 ns and a contact time of 0.01 ns. The temperature increase is negligible (Figure 13b), so eq 6 applies. These results suggest that out-of-plane vibrations are even more effective for lifting off ice clusters than water droplets, requiring less power for detachment (compare with Figure 6d).

## Conclusions

Within these studies, atomic-scale molecular dynamics simulations were performed to analyze the effectiveness of the kinetic energy transfer from a vibrating substrate to water and ice agglomerates placed on its surface. Numerical modeling has aimed at better understanding the mechanism of kinetic energy transfer from the substrate to ice/water. This analysis has provided useful information to design the best conditions for water removal and deicing systems based on the use of surface acoustic waves (SAW). In deicing systems based on SAWs, the ratio between a longitudinal and transverse component of the Rayleigh wave can be controlled by the elastic properties of the substrate (Poisson ratio), anisotropy of the material as well as the wave actuation conditions. In this regard, the results obtained in the previous analysis have revealed that the energy transfer and activation of water and ice are not affected only by the substrate vibrating modes. Specifically, we prove that the ice activation modes can be modified by tuning the substrate surface properties toward the reduction of ice adhesion, a characteristic that is indirectly related to the substrate wettability (hydrophobicity or hydrophilicity).

The results of atomic-scale simulations performed within these studies have enabled us to identify three phenomena responsible for water removal from the surface, namely, atomization, boiling, and lift-off. However, it has been found that for hydrophilic surfaces, lift-off processes are predictable only for a large mechanical energy transfer. Simulations also support that the transverse component of the SAWs (out-of-plane vibrations) would be more efficient for the energy transfer to water, resulting in more efficient energy-transfer phenomena as compared with the effect of a longitudinal component (in-plane vibrations). The results also show that the water removal mechanisms are different for hydrophilic and hydrophobic surfaces. In the first case, the dominant process is water atomization (evaporation), which transforms into boiling for larger-vibration amplitudes. In the hydrophobic case, the water droplet rolls over the surface and lifts off for higher vibrational energies.

Energy transfer from the vibrating substrate to water and ice is different since liquid accommodates strain by plastic deformation and the ice is not easily deformed. The results of molecular dynamics simulations indicate that both in-plane and out-of-plane vibrations are more effective in the case of ice. This effect is confirmed by the molecular dynamics studies performed herewith. It can be found that in-plane vibrations are much less effective than out-of-plane vibrations, especially for larger vibrational amplitudes. Larger amplitude in-plane vibrations result in ice melting for the hydrophilic surface, which is not the case for the hydrophobic one. On the other hand, for out-of-plane vibrations, the ice on the hydrophobic surface melts at lower amplitude while for larger amplitude it rapidly lifts off of the surface.

The above-mentioned results from our simulation analysis can be translated into some practical guidelines for the experimental design of an active, SAW-based, deicing system:

1. The selection of the surface state as well as the design of SAW actuators should be oriented to amplify the out-of-plane wave component of the vibration since it is more effective for both anti-icing and deicing processes.
2. The hydrophobic state of the surface facilitates the melting of ice even for relatively low amplitudes of out-of-plane vibrations. For hydrophobic surfaces, the in-plane vibrations are insufficient to cause the melting of the whole ice cluster.
3. Hydrophilic surfaces and large amplitudes facilitate the melting of ice for the in-plane component of SAW when larger amplitudes are applied.
